# Design of Higher-*k* and More Stable Rare Earth Oxides as Gate Dielectrics for Advanced CMOS Devices

**DOI:** 10.3390/ma5081413

**Published:** 2012-08-17

**Authors:** Yi Zhao

**Affiliations:** School of Electronic Science and Engineering, Nanjing University, Nanjing 210093, China; E-Mail: yzhao@nju.edu.cn

**Keywords:** gate dielectrics, hafnium oxide, lanthanum oxide, ternary oxides, permittivity, crystallization temperature, hygroscopic tolerance

## Abstract

High permittivity (*k*) gate dielectric films are widely studied to substitute SiO_2_ as gate oxides to suppress the unacceptable gate leakage current when the traditional SiO_2_ gate oxide becomes ultrathin. For high-*k* gate oxides, several material properties are dominantly important. The first one, undoubtedly, is permittivity. It has been well studied by many groups in terms of how to obtain a higher permittivity for popular high-*k* oxides, like HfO_2_ and La_2_O_3_. The second one is crystallization behavior. Although it’s still under the debate whether an amorphous film is definitely better than ploy-crystallized oxide film as a gate oxide upon considering the crystal boundaries induced leakage current, the crystallization behavior should be well understood for a high-*k* gate oxide because it could also, to some degree, determine the permittivity of the high-*k* oxide. Finally, some high-*k* gate oxides, especially rare earth oxides (like La_2_O_3_), are not stable in air and very hygroscopic, forming hydroxide. This topic has been well investigated in over the years and significant progresses have been achieved. In this paper, I will intensively review the most recent progresses of the experimental and theoretical studies for preparing higher-*k* and more stable, in terms of hygroscopic tolerance and crystallization behavior, Hf- and La-based ternary high-*k* gate oxides.

## 1. Introduction

High permittivity (*k*) gate dielectric films are widely studied to substitute SiO_2_ as gate oxides to suppress the unacceptable gate leakage current when the traditional SiO_2_ gate oxide becomes ultrathin. However, it is well known that some rare earth high-*k* oxides, especially La_2_O_3_, are not very stable in air and very hygroscopic, forming hydroxide [1,2]. As a gate dielectric, it is inevitable that it becomes involved with wet processes (water is used) and exposure to air in the conventional CMOS process [3]. Therefore, before we consider the possibility of rare earth oxides as a high-*k* gate dielectric, it is necessary to investigate the effects of moisture absorption on the properties of the La_2_O_3_ film. If the moisture absorption can degrade the properties of rare earth oxides as high-*k* gate dielectric, it will be very important to clarify the mechanisms of the moisture absorption to propose methods for stabilizing rare earth oxide films in air via suppressing the moisture absorption. Furthermore, one very important reason for lanthanum oxide (La_2_O_3_) as a promising high-*k* gate dielectric to replace SiO_2_ is its high permittivity. However, many low permittivity La_2_O_3_ films have also been reported [4,5,6].

In this paper, I will intensively review the most recent progresses of the experimental and theoretical studies for preparing higher-*k* and more stable, in terms of hygroscopic tolerance and crystallization behavior, Hf- and La-based ternary high-*k* gate oxides.

## 2. Design for More Stable High-*k* Gate Dielectric Films

**Figure 1:**
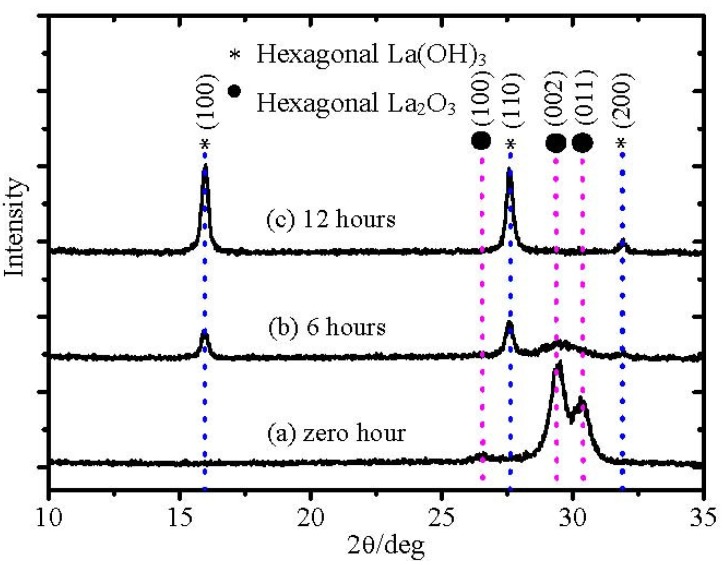
XRD patterns of La_2_O_3_ films on silicon after exposure to air for: (**a**) zero hours; (**b**) 6 hours and (**c**) 12 hours.

**Figure 2:**
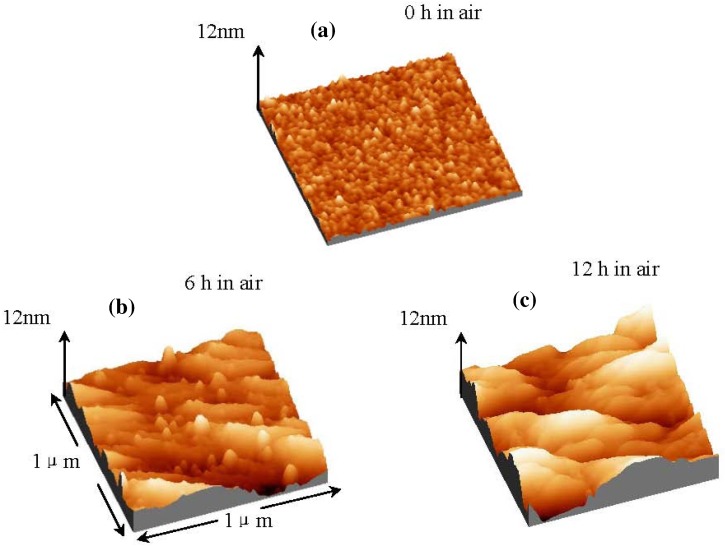
AFM images (1 μm × 1 μm) of La_2_O_3_ film surfaces after exposure to air for: (**a**) zero hour; (**b**) 6 hours and (**c**) 12 hours.

Figure 1 shows the X-ray diffraction (XRD) patterns of La_2_O_3_ films exposed to air over different times. From the XRD pattern, it is found that La_2_O_3_ film with zero hour exposed to air is poly-crystallized in the hexagonal phase. After exposure to air for 6 hours, a couple of peaks attributed to La(OH)_3_ appear, whereas the intensities of peaks attributed to the hexagonal La_2_O_3_ decrease. After exposure to air for 12 hours, strong La(OH)_3_ phase peaks are found, while peaks of hexagonal La_2_O_3_ disappear completely. Therefore, we can conclude that the amount of hexagonal La(OH)_3_ in the La_2_O_3_ film increased with the time exposed to air. Figure 2 shows atomic force microscopy (AFM) images of La_2_O_3_ films after exposure to air for different times: 0, 6, and 12 hours. It can be obviously observed that the surface roughness of La_2_O_3_ films increases with time exposed to air, from 0.5 nm to 2.4 nm. In terms of the reason for the surface roughness enhancement after the moisture absorption, one very possible reason is non-uniform moisture absorption of the La_2_O_3_ film, followed by non-uniform volume expansion of the film due to moisture absorption. A slight film thickness increase after the moisture absorption could be observed (data is not shown here), which indicates the volume expansion of the film. The original cause of the volume expansion is the density difference between hexagonal La(OH)_3_ and hexagonal La_2_O_3_. The density of hexagonal La(OH)_3_ (ρ = 4.445 g/cm^3^) [7,8] is much smaller than that of hexagonal La_2_O_3_ (ρ = 6.565g/cm^3^).

## 3. Low Permittivity Phenomena of La_2_O_3_ Films

**Figure 3:**
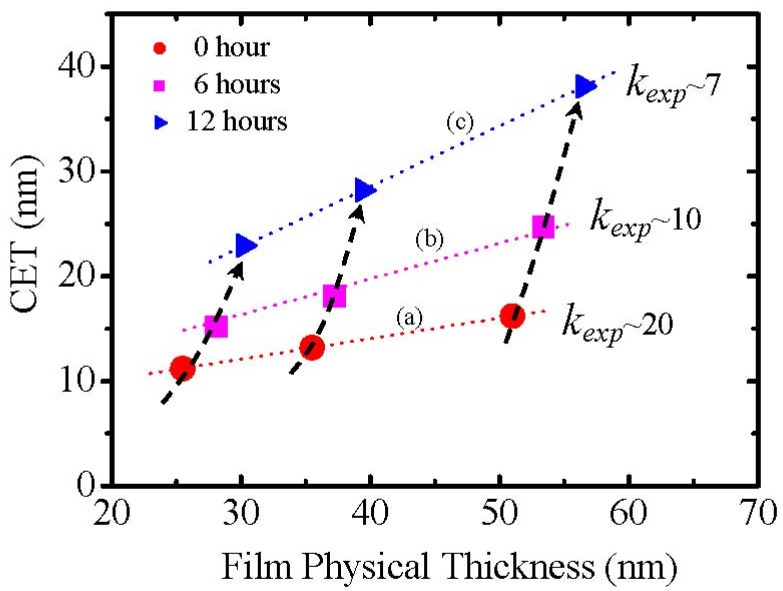
The relationship of CET to La_2_O_3_ physical thickness for Au/SiO_2_/La_2_O_3_/Si MIS capacitors. The sample was exposed to air for: (**a**) zero hours; (**b**) 6 hours and (**c**) 12 hours before SiO_2_ layer deposition.

In terms of the reasons for the low permittivity of La_2_O_3_ films reported in literature, one very possible reason could be moisture absorption due to the formation of lanthanum hydroxide as discussed above. Figure 3 shows a CET (capacitance equivalent thickness) *versus* a La_2_O_3_ film thickness plot for different samples. The zero hour exposure to air means that the sample was put in the sputtering chamber for SiO_2_ layer deposition as quickly as possible after annealing in a rapid thermal annealing (RTA) furnace. Also, the permittivity (*k_exp_*, k-value obtained experimentally from the slope) of the La_2_O_3_ film exposed to air can be calculated from the slope of linear fitting to experimental CETs. It is observed that the permittivity of the film is degraded with time exposed to air (Figure 3). The permittivity of La_2_O_3_ film in air for 0 hour is about 20. And exposure to air for 12 hours, the permittivity is degraded to only 7.

As discussed earlier, it has been concluded that the amount of hexagonal La(OH)_3_ in the La_2_O_3_ film increases with time exposed to air. Although there is no report about the permittivity of hexagonal La(OH)_3_, we can estimate the permittivity of hexagonal La(OH)_3_ on the basis of an additivity rule of the polarizability from Shannon’s consideration [7]. From the **Clausius-Mossotti** relationship, the dielectric constant is described by:

*k* = (3V_m_ + 8πα^T^)/(3V_m_ − 4πα^T^) (1)

where V_m_ and α^T^ denote molar volume and total polarizability respectively. For hexagonal La(OH)_3_, α^T^ is 12.81 Å^3^ from Shannon’s additivity rule [7], (α^T^(La(OH)_3_) = α(La^3+^) + 3α(OH^−^)) and V_m_ is 71 Å^3^ from Reference [8]. With the above values, we can estimate the permittivity of hexagonal La(OH)_3_ which is about 10. This result indicates that hexagonal La(OH)_3_ has a much lower permittivity compared to La_2_O_3_. Therefore, the effective permittivity of La_2_O_3_ film exposed to air could be degraded. In fact, with time exposed to air, Figure 3 shows the degradation of *k_exp_* (*k*-value obtained experimentally from the slope), though it is necessary to take account of an inhomogeneity of the film due to the partial reaction of the La_2_O_3_ with moisture. Therefore, the moisture absorption which causes the formation of low permittivity lanthanum hydroxide should be a very possible reason for scattering of the permittivity value of La_2_O_3_ films in previous literatures [4,5,6], although details of the process are not mentioned in the literature.

From Figure 1, it could be concluded that with time exposed to air, the amount of hexagonal La(OH)_3_ in La_2_O_3_ film increases and then the density of the film is degraded. Therefore, the effect of moisture absorption on the surface roughness should be another concern of hygroscopic La_2_O_3_ film application. According to the above discussion, it seems that the reported low permittivity of the La_2_O_3_ can be attributed to moisture absorption phenomena. However, we also have to note that the permittivity of La_2_O_3_ film in air for 0 hour was still a little low, about 20. This value is much lower than the reported highest one, 27 [9], although the possibility of moisture absorption still cannot be excluded, because the sample was exposed to the air. To exclude the effect on the permittivity of La_2_O_3_ films and obtain the permittivity of La_2_O_3_ films without moisture absorption, we used the *in-situ* heating method in a high vacuum chamber. The La_2_O_3_ film was annealed at 400 °C in the high vacuum (HV) chamber (10^−6^ Pa) to make lanthanum hydroxide decompose into La_2_O_3_ and H_2_O and then followed by 6 nm SiO_2_ layer deposition to prevent moisture absorption after removal from the sputtering chamber for the electrode deposition.

**Figure 4:**
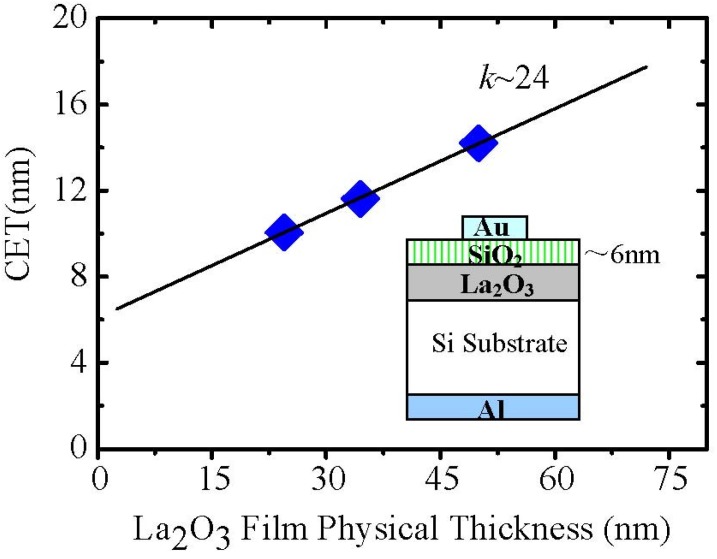
The relationship of CET to the La_2_O_3_ physical thickness for Au/SiO_2_/La_2_O_3_/Si/Al MIS capacitors.

Capacitance-voltage (C-V) measurements were performed for the Au/SiO_2_/La_2_O_3_/Si/Al metal insulator semiconductor (MIS) capacitors with a frequency of 100 kHz. The capacitance equivalent thickness (CET) has a good linear relationship with La_2_O_3_ film thickness, as shown in Figure 4, where the CET includes both La_2_O_3_ and SiO_2_films. Here, note that the thickness of capping SiO_2_ layer was fixed (~6 nm) and the thickness of La_2_O_3_ film was varied. Then, the permittivity of La_2_O_3_ can be calculated from the slope to be about 24. This result obviously indicates that the permittivity of our La_2_O_3_ film is still a little low, even though moisture absorption by the film was prevented. This means that moisture absorption is not the only factor contributing to the low permittivity of La_2_O_3_ films.

### 3.1. Hygroscopic Tolerance Enhancement of La_2_O_3_ Films

As discussed earlier, the moisture absorption process of La_2_O_3_ films is related with the formation of the OH ion. In the XRD pattern, peaks of hexagonal La(OH)_3_ appeared after exposure to air for 6 hours (Figure 1). Based on the consideration of possible reactions of the moisture absorption of La_2_O_3_ films, one very possible mechanism is the intrinsic reaction of La_2_O_3_ and H_2_O.

Due to the high ionicity of La_2_O_3_, it can react with H_2_O directly as per the following Equations:

La_2_O_3_ → 2La^3+^ + 3O^2−^ (2)

3H_2_O +3O^2‒^ → 6OH^−^ (3)

This moisture absorption progress is mainly due to the small lattice energy of La_2_O_3_ that promotes the reaction [10]. Lattice Energy (U) is the energy required to completely separate one mole of a solid ionic compound into gaseous ions which indicates the strength of the ionic bonds in an ionic lattice as shown below:

*M_m_X_n_* ⇒ *mM^n+^* + *nX^m−^* (4)

It has been reported that the lattice energy of ionic oxides is inversely proportional to the sum of the metal ion and oxygen ion radius [11]. In other words, the oxide with a larger metal ion radius shows a smaller lattice energy. In the case for rare earth oxides, because lanthanum ions have the largest radius, La_2_O_3_ shows the smallest lattice energy within rare earth oxides [12].

Thus, to enhance the hygroscopic tolerance of La_2_O_3_ films, it is necessary to enhance the lattice energy of La_2_O_3_. Furthermore, poorly crystallized film is looser than well crystallized. This makes water easier to diffuse into the film and react with La_2_O_3_. Therefore, one method to enhance the hygroscopic tolerance is to enhance the crystallinity of La_2_O_3_ film. As the poor crystallinity is intrinsic to La_2_O_3_, to enhance the crystallinity of La_2_O_3_, doping with other elements or oxides is necessary. When we select oxides for doping, we have to consider the lattice energy, and larger lattice energy oxides are preferred. From the phase diagram of the La_2_O_3_-Y_2_O_3_ system [13], a high melting point of La_2−*x*_Y*_x_*O_3_ can be observed, which indicates a low crystallization temperature of La_2−*x*_Y*_x_*O_3_. On the other hand, Y_2_O_3_ shows a much lower crystallization temperature than La_2_O_3_. It is very possible that La_2−*x*_Y*_x_*O_3_ films could also exhibit a low crystallization temperature or be very easy to be crystallized [14]. Furthermore, Y is in the same element group in the elements table as La and is the nearest element to La. It can be expected that La_2−*x*_Y*_x_*O_3_ might show similar properties as La_2_O_3_: for example permittivity, large band gap, and so on.

La_2−*x*_Y*_x_*O_3_ films with different Y atomic concentrations (Y/La + Y = 0%, 10%, 40%, 70%, 90% and 100%) were deposited on the HF-last Si (100) substrates or thick Pt films deposited on SiO_2_/Si substrates by RF co-sputtering of La_2_O_3_ and Y_2_O_3_ targets (provided by Kojundo Chemical, Saitama, Japan) in Ar ambient at room temperature and then annealed at 600 °C in pure N_2_ or 0.1%-O_2_+N_2_ ambient for 30 seconds in a rapid thermal annealing (RTA) furnace. The Y concentrations were determined by x-ray photoelectron spectroscopy (XPS) measurement. Moisture absorption experiments were performed in room air. The temperature and relative humidity of the air was 25 °C and 25% respectively. The XRD patterns of films before and after the moisture absorption were investigated. The MIM (metal-insulator-metal) capacitors on thick Pt films deposited on SiO_2_/Si substrates were prepared by depositing the Au film on the La_2−*x*_Y*_x_*O_3_ films to evaluate the permittivities. Au was also deposited on some La_2_O_3_ and La_2−*x*_Y*_x_*O_3_ films on silicon to form Au/La_2_O_3_ or La_2−*x*_Y*_x_*O_3_/Si metal insulator semiconductor (MIS) capacitors. The capacitance-voltage (C–V) with a frequency of 100 kHz and the gate current density-gate voltage (J–V) measurements were performed for MIS capacitors. The physical thicknesses of films were determined with grazing incident x-ray reflectivity (GIXR) and spectroscopic ellipsometry (SE) measurements.

**Figure 5:**
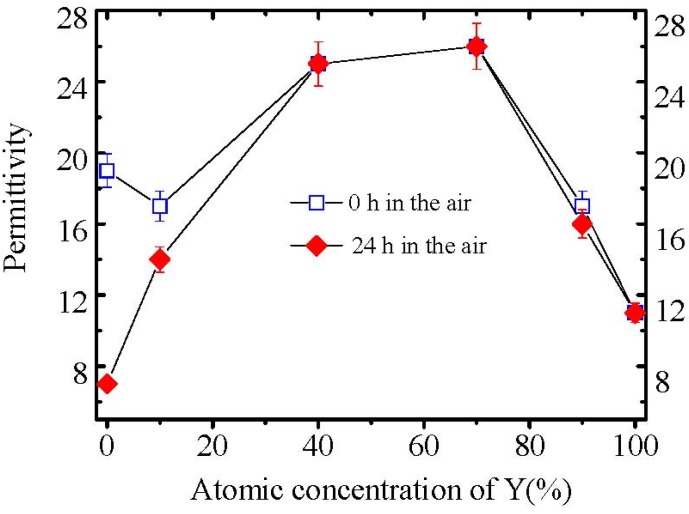
Variation of the permittivities of La_2−*x*_Y*_x_*O_3_ films with Y concentration. The permittivities were determined by MIM capacitors.

**Figure 6:**
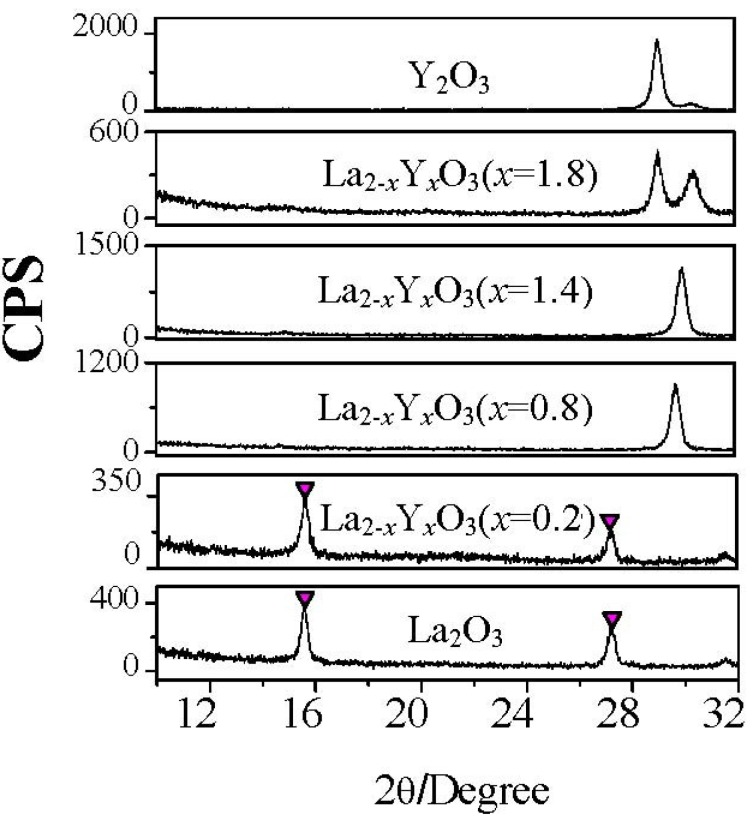
XRD patterns of La_2−*x*_Y*_x_*O_3_ films with different Y concentrations after exposure to air for 24 hours. Temperature and relative humidity of the air is 25 °C and 50% respectively. The films were annealed at 600 °C. ( 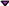: hydroxide).

Figure 5 shows the permittivities of all La_2−*x*_Y*_x_*O_3_ films after exposed to air for 0 and 24 hours. No permittivity degradation of La_2−*x*_Y*_x_*O_3_ (*x* = 0.8), La_2−*x*_Y*_x_*O_3_ (*x* = 1.4), La_2−*x*_Y*_x_*O_3_ (*x* = 1.8) and Y_2_O_3_ films was observed after films were exposed to air for 24 hours. However, the permittivities of La_2−*x*_Y*_x_*O_3_ (*x* = 0.2) film and La_2_O_3_ film decrease dramatically after exposure to air for 24 hours, due to the formation of low permittivity hydroxide (Figure 6). The XRD patterns of all La_2−*x*_Y*_x_*O_3_ films exposed to air for 24 hours are shown in Figure 6. The characteristic peaks attributed to hexagonal hydroxide due to the moisture absorption appear in XRD patterns of La_2−*x*_Y*_x_*O_3_ (*x* = 0.2) film and La_2_O_3_ film, while those are not found in XRD patterns of La_2−*x*_Y*_x_*O_3_ (*x* = 0.8), La_2−*x*_Y*_x_*O_3_ (*x* = 1.4), La_2−*x*_Y*_x_*O_3_ (*x* = 1.8), and Y_2_O_3_ films. This means that when the Y concentration is higher than, or equal to, 40% (*x* = 0.8), the La_2−*x*_Y*_x_*O_3_ film will exhibit good moisture resistance. From the electrical properties measurements, we can also know the strong moisture-resistance of La_2−*x*_Y*_x_*O_3_ films. No degradation of C–V characteristics of La_2−*x*_Y*_x_*O_3_ (*x* = 1.4) film is observed after exposed to air for 24 hours. At the same time, the gate leakage current of Au/La_2−*x*_Y*_x_*O_3_ (*x* = 1.4)/Si MIS capacitor shows no apparent increase after exposed to the air for 24 hours. On the contrary, for the La_2_O_3_ film after exposure to air for 24 hours, the maximum capacitance decrease in the accumulation side of the C–V curve and the flat band shift are observed. The gate leakage current of the Au/La_2_O_3_/Si MIS capacitor also increased by about two orders of magnitude when the La_2_O_3_ film was exposed to air for 24 hours before Au deposition.

As discussed earlier, the moisture absorption reaction is intrinsically due to the small lattice energy of La_2_O_3_. The larger lattice energy could induce stronger moisture resistance due to the suppression of a reaction between La_2_O_3_ and H_2_O. It is possible that the well crystallized film should exhibit a relatively larger lattice energy than the amorphous or poorly crystallized film. In our experiments, La_2_O_3_ had poorer crystallinity (full-width at half-maximum (FWHM) ≈ 1.4 degree) than 40%Y (*x* = 0.8) and 70%Y (*x* = 1.4) La_2−*x*_Y*_x_*O_3_ films (FWHM ≈ 0.4 degree) from the XRD patterns. This indicates that the lattice energy of 40%Y (*x* = 0.8) and 70%Y (*x* = 1.4) La_2−*x*_Y*_x_*O_3_ films might be larger than that of La_2_O_3_, thanks to better crystallinity. Furthermore, Y_2_O_3_ exhibits a much larger lattice energy of 158.47 eV/mol than that of La_2_O_3_ (146.83 eV/mol). Therefore, Y_2_O_3_ doping could effectively enhance the lattice energy of La_2_O_3_. Furthermore, the lattice energy is related to the crystal forms of the film. One thing we should note is that 40%Y (*x* = 0.8) and 70%Y (*x* = 1.4) La_2−*x*_Y*_x_*O_3_ films also show a much higher permittivity (~26) than La_2_O_3_ film in our study. The high permittivities are due to the formation of high permittivity hexagonal phase of La_2−*x*_Y*_x_*O_3_ films with very good crystallinity after the annealing. The permittivity of lanthanum based oxides will be discussed in more details in the later paragraphs. These results indicate that La_2−*x*_Y*_x_*O_3_ films not only show strong moisture resistance, also show a high permittivity when the Y concentration is between 40% (*x* = 0.8) and 70% (*x* = 1.4).

Therefore, due to the introduction of Y_2_O_3_, 40%Y (*x* = 0.8) and 70%Y (*x* = 1.4) La_2−*x*_Y*_x_*O_3_ films after annealed at 600 °C exhibit much larger lattice energy than La_2_O_3_ film which induces stronger hygroscopic tolerance of La_2−*x*_Y*_x_*O_3_ films. The results also indicate that phase control is an effective method to enhance the moisture-robustness of La_2_O_3_ films.

To further understand the mechanism for enhancing moisture resistance via second oxide doping, thermodynamic analysis of moisture absorption phenomena in high-*k* gate dielectrics has been performed [15]. Intrinsically, the moisture absorption phenomenon in high-*k* oxides is the reaction between the solid oxide (*M_m_O_n_*) film and gaseous state water (*H_2_O*) in air, which can be expressed by Equation (5) as discussed above.

*M_m_O_n_* + n*H_2_O*(*g*) ⇄ *M_m_*(*OH*)_2*n*_ (5)

It is well known that the rate of a chemical reaction can be indexed by the Gibbs free energy change, Δ*G*, of the reaction, which is given by Equation (6) [16].

Δ*G* = Δ*H* − *T*Δ*S* (6)

where, Δ*H* is the enthalpy change of the reaction, Δ*S* is the entropy change of the reaction, and *T* is the ambient temperature. Both of Δ*S* and Δ*H* are calculated by subtracting the sum (entropy or enthalpy) of the left side of the reaction equation to that of the right side of the reaction equation. The entropy and enthalpy data of *H*_2_*O*, *M*(*OH*) and *M_m_O_n_* were obtained from the database of HSC Chemistry software [17] and Reference [18] (only for Hf(OH)_4_). The negative Δ*G*, meaning the decrease in system energy after the reaction, indicates a possibility for the occurrence of the reaction. Furthermore, when Δ*G* is negative, a larger absolute value of Δ*G* means a larger reaction rate. However, note that in the real case of high-*k* oxide films, the reaction rate is influenced by many other factors. In our study, we focused on the thermodynamic process of the moisture absorption reaction, which could be the main factor for determining the reaction rate.

**Figure 7:**
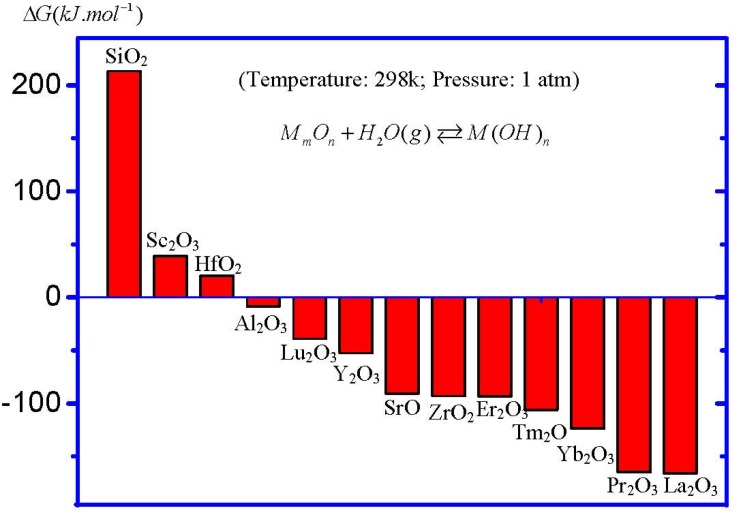
Δ*G* of the moisture absorption reactions in high-*k* oxides under standard conditions. All entropy and enthalpy data of oxides, H_2_O and hydroxides were obtained from the database of HSC chemistry software, except for Hf(OH)_4_, which is cited from Reference [18].

Figure 7 shows the calculated Δ*G* of the moisture absorption reactions of main high-*k* oxide candidates. For the purpose of comparison, the data of the reaction between SiO_2_ and H_2_O is also included in the figure. It can be obviously observed that, under standard conditions (temperature = 298.15 K, pressure = 1 atm), the moisture absorption reaction in SiO_2_ could not occur, since the Δ*G* of the reaction is positive. This fact is the chemical reason for the stable SiO_2_ film in the air as a gate oxide. On the other hand, a large range of Δ*G* values in high-*k* oxides, indicating different moisture-absorption-reaction rates, could be observed. Hafnium oxide (HfO_2_), the most studied high-*k* gate dielectric so far, shows a positive Δ*G*, meaning a small moisture-absorption-reaction rate. This result is coincident with the experimental results, since there have been few reports about the moisture absorption phenomenon in HfO_2_. On the contrary, note that zirconium oxide (ZrO_2_), which is also thought to be a promising high *k* oxide, shows a large negative Δ*G*. However, the moisture absorption phenomenon in ZrO_2_ film as a high *k* gate dielectric has not been emphasized in the literature yet. As a matter of fact, the formation of zirconium hydroxide at the surface of ZrO_2_ film has been reported [19]. Furthermore, La_2_O_3_ shows the most negative Δ*G* among all main high-*k* oxide candidates. This is the reason for the serious moisture absorption phenomenon in La_2_O_3_ films. This fact also suggests that the moisture absorption phenomenon in La_2_O_3_ films is the intrinsic property of La_2_O_3_, rather than caused by some external factors. On the other hand, it can be found from Figure 7 that all rare earth oxides show a large moisture-absorption-reaction rate, except for scandium oxide (Sc_2_O_3_), meaning that most pure rare earth oxides might not be suitable as high-*k* gate oxides, although they usually show high permittivities.

**Figure 8:**
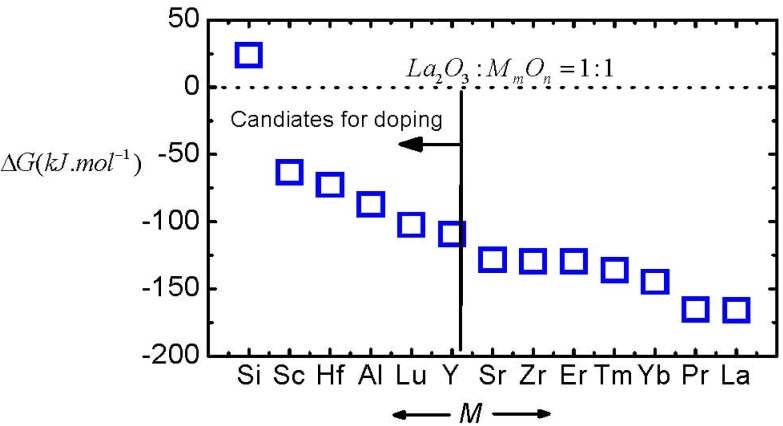
The Δ*G* of the moisture absorption reactions of La-based ternary oxides, calculated by averaging the Δ*G* of La_2_O_3_ and the second oxide. The molecule ratio between La_2_O_3_ and the second oxide in ternary oxides is 1:1.

Next, we discuss how to enhance the moisture resistance of rare earth oxides, especially that of La_2_O_3_. Through considering the thermodynamic process of the moisture absorption reaction as shown in Equation (5), the most direct method of enhancing the moisture resistance or decreasing the moisture-absorption-reaction rate of an oxide film is doping with a second oxide that exhibits a stronger resistance to moisture absorption. We have observed that Y_2_O_3_ doped La_2_O_3_ films show much stronger strong moisture resistance than La_2_O_3_ [20,21], which is a demonstration of this method (Figure 6). Figure 8 shows the Δ*G* of several La-based ternary oxides with a molecule ratio of 1:1 between La_2_O_3_ and the second oxide, which are simply calculated by averaging the Δ*G* of the moisture absorption reaction of La_2_O_3_ and the second oxides.

As shown in Figure 8, doping a second oxide is an effective method for decreasing moisture-absorption-reaction speed. Furthermore, SiO_2_, Sc_2_O_3_, HfO_2_, Al_2_O_3_, Lu_2_O_3_, and Y_2_O_3_ are better candidates than other oxides for doping to enhance the moisture resistance of La_2_O_3_. On the other hand, note that the permittivity of the doped La_2_O_3_ has to be considered when we select a second oxide. This issue will be discussed further later. Furthermore, the moisture resistance of an oxide film is also affected by several external factors, like the crystallinity [20] of the film and oxygen vacancies in the film [22], which will be discussed in more detail later. We can understand these behaviors by a more detailed analysis of the moisture absorption reaction. The reaction in Equation (5) could be divided into three steps as shown in Equations (7), (8) and (9).

*M_m_O_n_* ⇄ m*M*^*n*+^ + n*O*^2−^ (7)

*O*^2−^ + 2*H*_2_*O* ⇄ 4*OH*^−^ (8)

*M*^*n*+^ + n*OH* ⇄ *M*(*OH*)_*n*_ (9)

The Equations (7) and (8) are the key reactions for determining the rate of the whole moisture absorption reaction. Physically, the reaction rate of Equation (7) is determined by the lattice energy of the oxide, which is mainly determined by the ionicity (or electronegativity) of *M* ion and could also be affected by the crystallinity of the oxide in the case of a thin film. The larger electronegativity means a larger ionicity, resulting in a smaller lattice energy and a larger reaction rate of Equation (7). In fact, the Δ*G* results in Figure 7 coincide well with the reported electronegativity data [23]. On the other hand, the reaction Equation (4) is responsible for the formation of OH^−^, resulting in the formation of hydroxide after being combined with *M^n+^* (Equation (9)). The oxygen vacancies, however, can also induce the formation of OH^−^, which is the reason for the more serious moisture absorption phenomenon in oxygen-deficient La_2_O_3_ films. However, as shown in Figure 7 a thermodynamic process could be the main and intrinsic factor for determining the rate of the moisture absorption reaction.

In summary, the moisture absorption phenomena in main high-*k* gate oxides have been theoretically discussed by comparing the Gibbs free energy change of the moisture absorption reactions of these oxides. The results show that moisture absorption could occur in most high-*k* oxides, especially in rare earth oxides. On the other hand, La_2_O_3_ shows the largest moisture-absorption-reaction speed among main high-*k* oxide candidates. To enhance the moisture resistance of La_2_O_3_, doping a second oxide, which has a stronger moisture resistance than La_2_O_3_, could be an applicable solution.

### 3.2. Hygroscopic Tolerance Enhancement of La_2_O_3_ Films by Ultraviolet Ozone Treatment

In our experiments, we found that the oxygen-ambient-annealing La_2_O_3_ film shows stronger moisture resistance than nitrogen-ambient-annealing La_2_O_3_ film, although the moisture absorption phenomenon was still observed after being in air for several days. So, it seems that moisture absorption is partly related to oxygen vacancies in the films. In other words, if the oxygen vacancies in La_2_O_3_ film could be eliminated, moisture resistance could be enhanced to some degree. The most direct method is to eliminate or heal the oxygen vacancy. It has been reported that ultraviolet (UV) ozone treatment at room temperature can eliminate oxygen vacancies in oxide films [24]. Thus, moisture absorption suppression is expected with UV ozone post treatment, thanks to the healing of oxygen vacancies. The low temperature of UV ozone treatment merits the CMOS process which could prevent the formation of a thick interface layer. The interface layer could enhance the total EOT (Equivalent Oxide Thickness) of the gate dielectric. La_2_O_3_ films were deposited on HF-last Si by sputtering the La_2_O_3_ target in argon at ambient room temperature and then annealed at 600 °C in pure N_2_ or 0.1%-O_2_+N_2_ ambient for 30 seconds in a rapid thermal annealing (RTA) furnace. Some samples were treated with UV ozone for 9 minutes at room temperature.

The moisture absorption experiments were performed in room air. The temperature and relative humidity of the air were 25 °C and 25%, respectively. The root-mean-square (rms) surface roughnesses and XRD patterns of films before and after the moisture absorption were investigated. Au was also deposited on some La_2_O_3_ films on silicon to form Au/La_2_O_3_/Si metal insulator semiconductor (MIS) capacitors. The capacitance-voltage (C–V) with a frequency of 100 kHz and the gate current density–gate voltage (J_g_–V_g_) measurements were performed for MIS capacitors. The physical thickness films were determined with grazing incident x-ray reflectivity (GIXR) and spectroscopic ellipsometry (SE) measurements.

**Figure 9:**
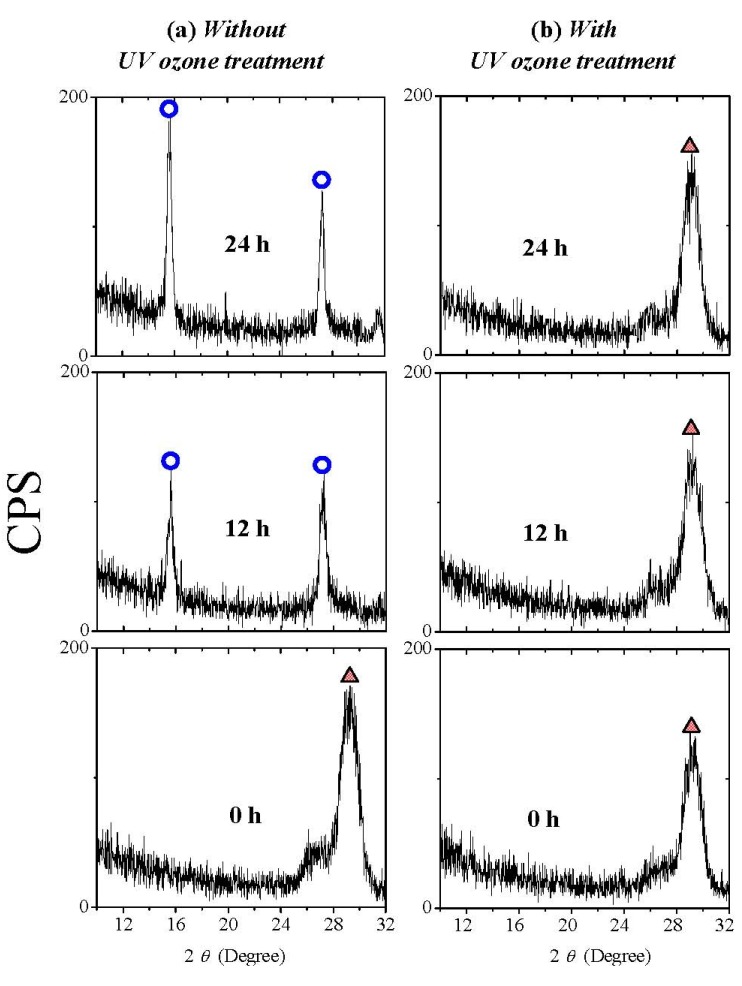
XRD patterns of La_2_O_3_ films (**a**) with and (**b**) without UV ozone post treatment after N_2_ annealing. Films were exposed to air (Temperature 25 °C and relative humidity about 25% respectively) for different times.

**Figure 10:**
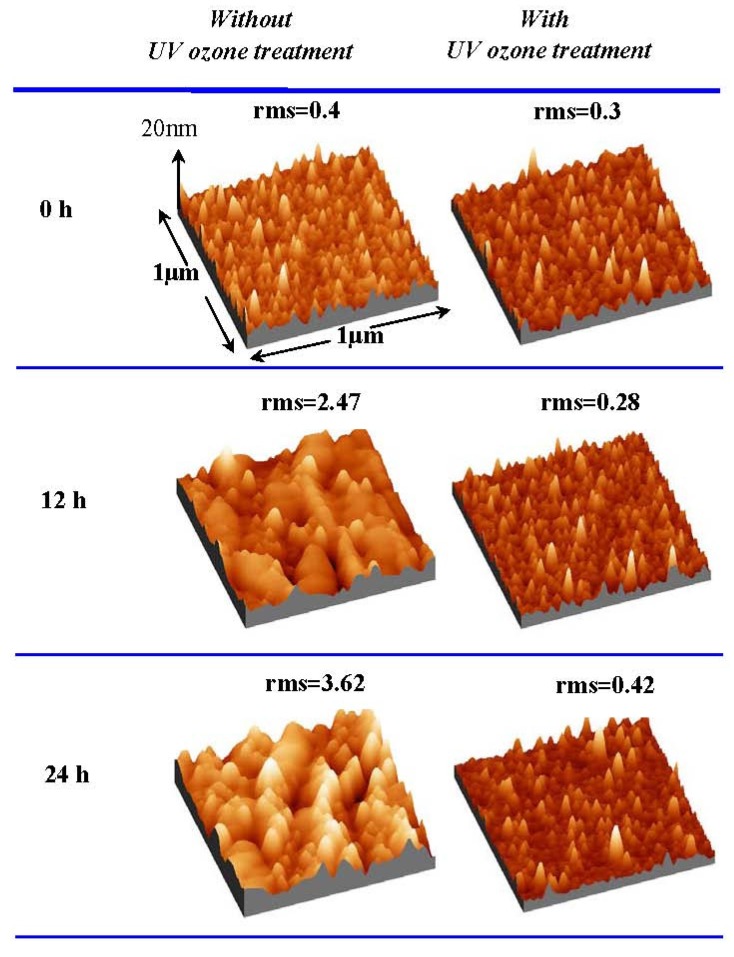
Surface AFM images (1 μm × 1 μm) of La_2_O_3_ films with and without UV ozone treatment after N_2_ annealing at 600 °C. Films were exposed to air for different times (Temperature and relative humidity of air: 25 °C and 25% respectively).

Since, as reported, that the UV ozone treatment can eliminate oxygen vacancies in the oxide films, moisture absorption suppression is expected with the UV ozone post treatment, thanks to the healing of oxygen vacancies. Figure 9 shows the XRD patterns of La_2_O_3_ films with and without UV ozone post treatment after N_2_ annealing. 0 hour in air means that the sample was measured as soon as possible after annealing or UV ozone post treatment. It is found that both are poly-crystallized in the hexagonal phase when they are exposed to air for 0 hour. After exposure to air for 24 hours, in the XRD pattern of the La_2_O_3_ film without UV ozone post treatment after N_2_ annealing, the characteristic peaks attributed to hexagonal La(OH)_3_ due to moisture absorption appear, while these peaks are not found in the XRD pattern of the La_2_O_3_ film with UV ozone post treatment. Figure 10 shows AFM images of La_2_O_3_ films with and without UV ozone post treatment after the films were exposed to air for different times. The root-mean-square (rms) surface roughness of the La_2_O_3_ film without UV ozone post treatment after N_2_ annealing increases with time exposed to air, due to the formation of low density hexagonal La(OH)_3_. In contrast, the surface roughness of the La_2_O_3_ film with UV ozone post treatment after N_2_ annealing increases very little even after the film was exposed to the air for 24 hours. The above results suggest that UV ozone treatment can suppress the moisture absorption of La_2_O_3_ films.

To investigate the origin of the suppression effect with UV ozone treatment, moisture resistances of La_2_O_3_ films with ambient oxygen (0.1%-O_2_ + N_2_) annealing and as-deposited La_2_O_3_ film (without annealing or post treatment) were also investigated. It was clearly observed (data is not shown here) that the rms surface roughness of the UV ozone post treatment film and ambient oxygen annealing film show almost no increase with the time exposed to air. On the contrary, as-deposited and N_2_ annealing films’ rms surface roughnesses rapidly increase with time exposure to air. Since UV ozone post treatment and ambient oxygen annealing cause the same effect of healing the oxygen vacancies, it is reasonable to think that the origin of the moisture absorption suppression with the UV ozone post treatment might be the healing of the oxygen vacancies in La_2_O_3_

As discussed previously, the hygroscopic phenomena in La_2_O_3_ films are due to the low lattice energy of La_2_O_3_. Therefore, it is considered that the oxygen vacancy can decrease the lattice energy of La_2_O_3_. The oxygen vacancy could enlarge the charge transfer between La and O atoms and then make the La-O bond more ionic, resulting in a smaller lattice energy of La_2_O_3_ films.

On the other hand, it means that if we can heal oxygen vacancies in the La_2_O_3_ films, moisture absorption should be suppressed to some degree. Ozone (O_3_) can enhance the kinetics of oxidation (or oxygen vacancy healing) compared with conventional thermal oxidation (ambient oxygen annealing). For the La_2_O_3_ films containing oxygen vacancies (La_2_O_3−*x*_), the oxidation reaction can occur at low temperatures, and can heal the oxygen vacancies in the La_2_O_3_ films during UV ozone treatment.

**Figure 11:**
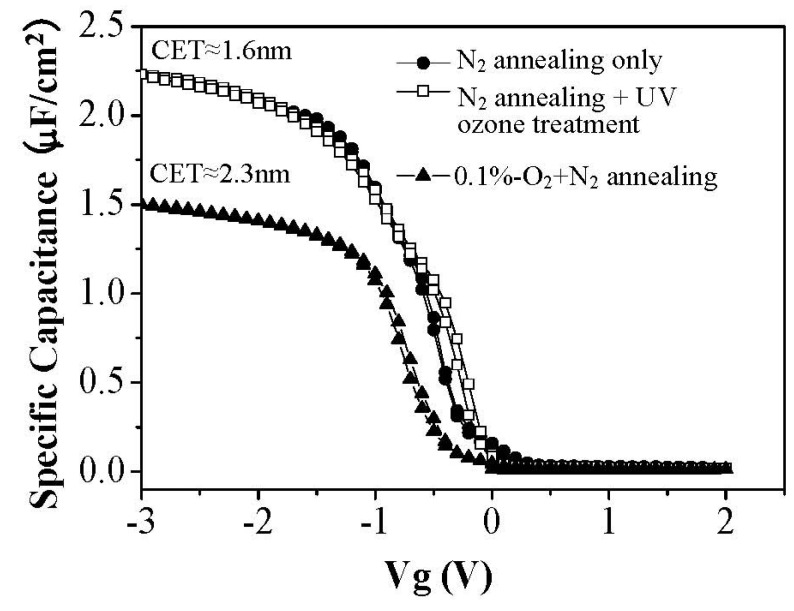
C–V curve (100 kHz) of Au/La_2_O_3_/Si MIS capacitors with and without UV ozone post treatment after N_2_ annealing.

On the other hand, for ambient oxygen annealing to heal oxygen vacancies, a high temperature process is generally necessary. Although ambient oxygen annealing shows similar effects as the UV ozone treatment in terms of the moisture absorption suppression, compared to the UV ozone post treatment, ambient oxygen annealing enhanced the capacitance equivalent thickness (CET) of the film (Figure 11) due to the formation of a thicker interface layer between silicon substrate and La_2_O_3_ film. Therefore, the UV ozone post treatment is a good method to suppress the moisture absorption suppression of La_2_O_3_ films with the merit of no interface layer thickness enhancement.

### 3.3. Design for Higher-*k* of HfO_2_ and La_2_O_3_ Gate Dielectric Films

One very important reason for lanthanum oxide (La_2_O_3_) as a promising high-*k* gate dielectric to replace SiO_2_ is its high permittivity. However, many low permittivity La_2_O_3_ films have been reported [4,5,6]. In terms of the reasons for the low permittivity of La_2_O_3_ films, two very possible ones are being considered as mentioned earlier. The first is moisture absorption which degrades the permittivity of La_2_O_3_ films due to the formation of low permittivity lanthanum hydroxide as discussed above [25]. The second is the low density of amorphous La_2_O_3_ films. In fact, the permittivity of La_2_O_3_ film without moisture absorption (0 hour in the air) still shows some low permittivity (~20). This indicates that the low permittivity could be an intrinsic property of La_2_O_3_ films, which could be partly attributed to poor cystallinity, *i.e*., not totally attributed to moisture absorption.

**Figure 12:**
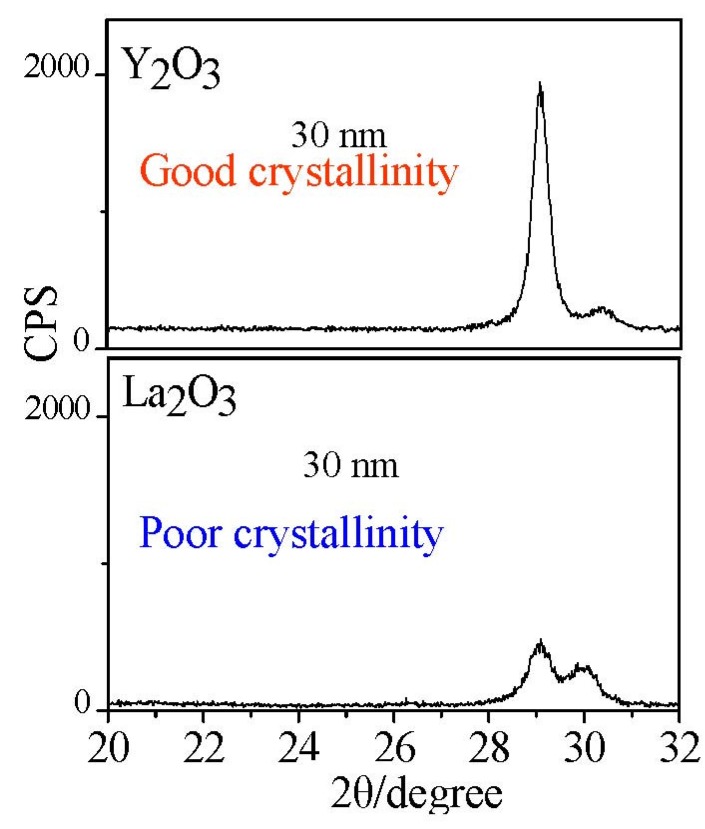
Cystallinity comparison of Y_2_O_3_ and La_2_O_3_ films.

Therefore, it is necessary to prepare well-crystallized La-based films to enhance and stabilize the permittivity of La_2_O_3_ films. From the phase diagram of the La_2_O_3_-Y_2_O_3_ system, a high melting point of La_2−*x*_Y*_x_*O_3_ could be observed which indicates a low crystallization temperature of La_2−*x*_Y*_x_*O_3_. On the other hand, Y_2_O_3_ shows a much lower crystallization temperature than La_2_O_3_ (Figure 12). It is very possible that La_2−*x*_Y*_x_*O_3_ films could also exhibit a low crystallization temperature. Furthermore, Y is in the same element group in the elements table as La and is the nearest element to La. It can be expected that La_2−*x*_Y*_x_*O_3_ can show similar properties as La_2_O_3_, for example permittivity, band gaps and so on, except for moisture absorption phenomena. On the other hand, a very common viewpoint is that amorphous film (high crystallization temperature) is better than crystallized film as high-*k* gate insulators. It is believed that grain boundaries in polycrystalline films might constitute electrical leakage paths, giving rise to dramatically increased gate leakage currents. However, there are few reports about the grain boundary induced leakage current in high-*k* gate dielectrics; currently, expitaxial (crystalline) film are also technologically feasible.

Among La-based high-*k* materials, La_1−*x*_Hf*_x_*O*_y_* and LaAlO_3_ are two attractive ones because La_1−*x*_Hf*_x_*O*_y_* is a good amorphous insulator up to 900 °C [26] and LaAlO_3_ shows a high permittivity and a large band gap. [19] However, La_1−*x*_Hf*_x_*O*_y_* film crystallizes in the pylochlore La_2_H_2_fO_7_ after annealing at 1000 °C [27], while in the conventional complementary metal-oxide semiconductor (CMOS) process, annealing higher than 1000 °C is necessary to activate the source and drain dopant. In terms of LaAlO_3_ film, as low permittivity LaAlO_3_ films (<20) are always reported, [28] it might be very difficult to prepare high permittivity LaAlO_3_ films. A very possible reason for the low permittivity of LaAlO_3_ films is the poor crystallinity which induces the low density of films. These results indicate that it is very difficult to prepare an amorphous high permittivity dielectric film as an alternative gate insulator. Although Ta_2_O_5_ film shows a high permittivity even in the amorphous state, [29] due to its very small conduction band offset with silicon, it cannot be used as a high-*k* gate dielectric. It is well known that La_2_O_3_ has a large conduction band offset with silicon of about 2.3 eV and a high permittivity. Therefore, La_1−*x*_Ta*_x_*O*_y_* film with an appropriate Ta concentration might be suitable as a gate dielectric which exhibits a medium conduction band offset with silicon, due to the introduction of La_2_O_3_. At the same time, a high permittivity of La_1−*x*_Ta*_x_*O*_y_* film can be expected thanks to the high permittivity of La_2_O_3_ and Ta_2_O_5_. In terms of crystallization temperature, due to the low melting point of La_1−*x*_Ta*_x_*O*_y_* from the La_2_O_3_-Ta_2_O_5_ phase diagram [30], La_1−*x*_Ta*_x_*O*_y_* films might show a high crystallization temperature. Therefore, we also investigated La_1−*x*_Ta*_x_*O*_y_* films with different Ta concentrations as high-*k* gate insulators in terms of the crystallization temperature, permittivity, band gap and electrical properties. The above discussion indicates that, theoretically and practically, both amorphous and well-crystallized high-*k* films might also be possible choices as gate insulators.

The La_2−*x*_Y*_x_*O_3_ and La_1-*x*_Ta*_x_*O*_y_* films with different Y or Ta atomic concentrations were deposited on the HF-last Si (100) substrates or thick Pt films deposited on SiO_2_/Si substrates by RF co-sputtering of La_2_O_3_ and Y_2_O_3_ or Ta_2_O_5_ targets (provided by Kojundo Chemical, Japan) in Ar ambient at room temperature. The Y and Ta concentrations were determined by x-ray photoelectron spectroscopy (XPS) measurement. The physical thicknesses of the films were determined with spectroscopic ellipsometry (SE) and glazing incident x-ray reflectivity (GIXR) measurements. The crystallinity of films was investigated by x-ray diffraction (XRD) measurement. The MIM (metal-insulator-metal) capacitors on thick Pt films deposited on SiO_2_/Si substrates were prepared by depositing the Au film on the La_2−*x*_Y*_x_*O_3_ or La_1−*x*_Ta*_x_*O*_y_* films to evaluate the permittivities. Au was also deposited on some La_2−*x*_Y*_x_*O_3_ and La_1−*x*_Ta*_x_*O*_y_* films on silicon to form Au/La_2−*x*_Y*_x_*O_3_ or La_1−*x*_Ta*_x_*O*_y_*/Si metal insulator semiconductor (MIS) capacitors. The capacitance-voltage (C-V) with a frequency of 100 kHz and gate current density-gate voltage (J-V) measurements were performed for the Au/La_2−*x*_Y*_x_*O_3_ or La_1−*x*_Ta*_x_*O*_y_*/Si MIS capacitors.

**Figure 13:**
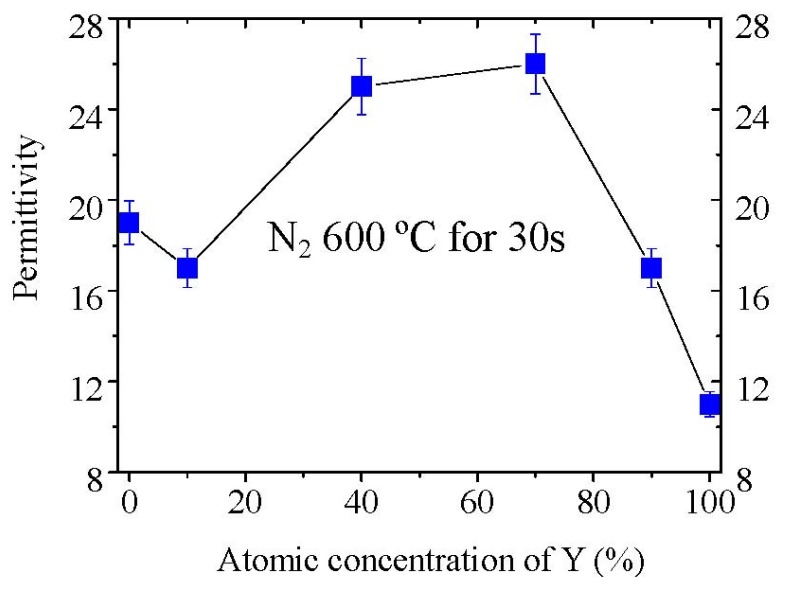
Variation of the permittivities of La_2−*x*_Y*_x_*O_3_ films with the Y concentration. Permittivities were determined by MIM capacitors. The films were thought to be exposed to the air for 0 hour rather than moisture prevented because we did not exclude the films from moisture on purpose, and just deposited the films with the Au electrode as quickly as possible after annealing in the RTA furnace.

Figure 13 shows the permittivity variation of La_2−*x*_Y*_x_*O_3_ films annealed at 600 °C in pure N_2_ ambient with the Y concentration. It is noticed that the permittivity of La_2_O_3_ film is low compared with the large value of 27 reported previously. The low permittivity of La_2_O_3_ film might be attributed to the poor crystallization of the film and to the moisture absorption because we did not intentionally exclude the sample from moisture. In our study, the Y_2_O_3_ film has a permittivity of 12 as reported [31]. The permittivity of La_2−*x*_Y*_x_*O_3_(*x* = 0.2) film is a little smaller than that of the La_2_O_3_ film, whereas the La_2−*x*_Y*_x_*O_3_(*x* = 0.8) and La_2−*x*_Y*_x_*O_3_(*x* = 1.4) films show much higher permittivity (~26) than La_2_O_3_ film in our study. This value is also very close to the high permittivity value of La_2_O_3_ film as reported [9]. When the Y concentration is as high as 90% (*x* = 1.8), the permittivity of La_2−*x*_Y*_x_*O_3_ film decreases to 15. But this value is still higher than the permittivity of Y_2_O_3_.

To explain the reason for high permittivity of La_2−*x*_Y*_x_*O_3_ films, it is necessary to discuss the **Clausius-Mosotti** equation for the theory calculation of permittivity [32].

**Figure 14:**
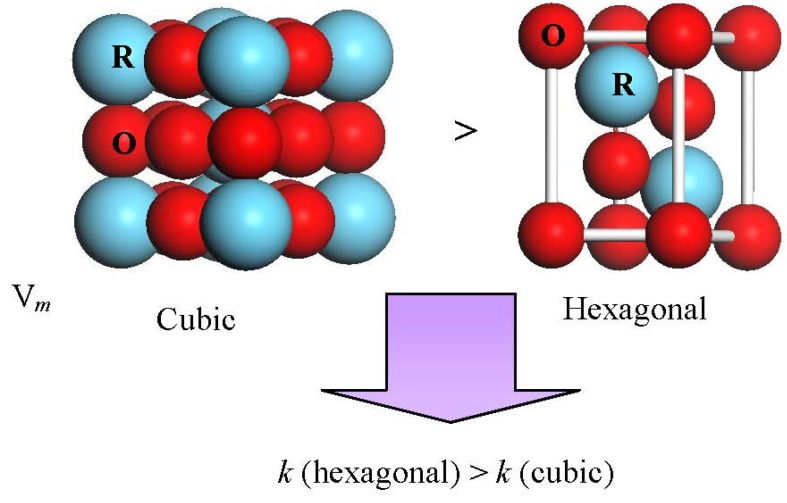
Molar volume comparison of hexagonal and cubic rare earth oxide (R_2_O_3_).

**Figure 15:**
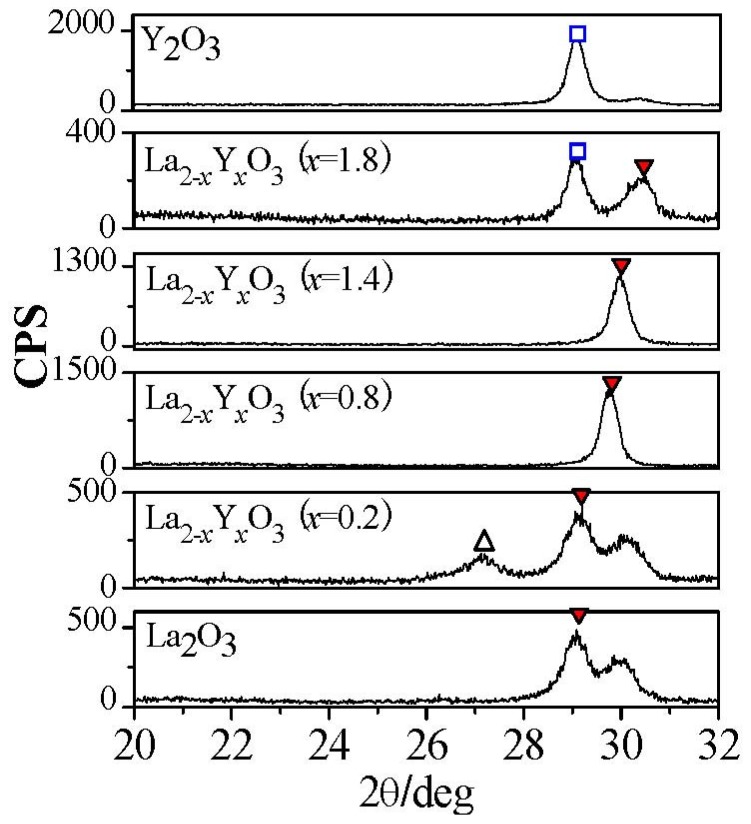
XRD patterns of La_2−*x*_Y*_x_*O_3_ films with different Y concentrations after annealing at 600 °C. (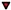: Hexagonal La_1−*x*_Y*_x_*O_3_ (002), : Cubic Y_2_O_3_ (222), 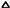: Cubic La_2_O_3_ (222)).

Put simply, the **Clausius-Mosotti** equation tells us that the permittivity of a well crystallized film is determined by the molar volume and total polarizability which is given as Equation (1). We can understand easily from Equation (1) that if α^T^ is assumed to be a constant in spite of the V_m_ change, the smaller V_m_ will induce a larger permittivity. For rare earth oxides (R_2_O_3_), the hexagonal phase exhibits much smaller molar volumes, as shown in Figure 14. For hexagonal La_2_O_3_, α^T^ is 18.17Å from the Shannon’s additivity rule (α^T^(La_2_O_3_) = 2α(La^3+^) + 3α(O^−2^)) and V_m_ is 82.7 Å^3^ from Reference [33]. With the above values, we can estimate that the permittivity of hexagonal La_2_O_3_ is about 35, which is larger than the reported permittivities of La_2_O_3_ films. This difference comes from the poor crystallinity of the reported La_2_O_3_ films and the low permittivity cubic phase of some La_2_O_3_ films. The same method is applied to hexagonal Y_2_O_3_ to estimate the permittivity. The V_m_ of hexagonal Y_2_O_3_ is assumed to be 90% to that of cubic Y_2_O_3_ [34], as in the case of La_2_O_3_, because no XRD pattern of the hexagonal Y_2_O_3_ has been reported. We can then estimate that the permittivity of hexagonal Y_2_O_3_ is 22, which is much larger than the permittivity of the cubic phase Y_2_O_3_ in our study (*k*~11). In summary, for rare earth oxides, hexagonal phase (and well crystallized) is preferred in order to achieve high permittivity. Next, let me explain the reason for the high permittivity of La_2−*x*_Y*_x_*O_3_ films. Figure 15 shows the XRD patterns of all La_2-*x*_Y*_x_*O_3_ films on Pt film after annealing at 600 °C. It can be observed that the La_2_O_3_ film is polycrystallized in the hexagonal phase. In the XRD pattern of La_2−*x*_Y*_x_*O_3_(*x* = 0.2) film, both peaks attributed to the cubic phase and hexagonal phase are found. Therefore, the permittivity of La_2−*x*_Y*_x_*O_3_(*x* = 0.2) film is smaller than that of the La_2_O_3_ film, due to the low permittivity of the cubic phase.

The 40%Y (*x* = 0.8) and 70%Y (*x* = 1.4) La_2−*x*_Y*_x_*O_3_ films are well crystallized in the hexagonal phase after annealing at 600 °C. The crystallinity of the film can be estimated with the full-width at half-maximum (FWHM) of the XRD peak. The smaller FWHM indicates better crystallinity. The FWHM of hexagonal (002) peak of 40%Y (*x* = 0.8) and 70%Y (*x* = 1.4) La_2−*x*_Y*_x_*O_3_ films’ XRD patterns are only 0.4 degree, while that of La_2_O_3_ film is about 1.4 degree. It indicates that 40%Y (*x* = 0.8) and 70%Y (*x* = 1.4) La_2−*x*_Y*_x_*O_3_ films exhibit a better crystallinity than La_2_O_3_ film. As reported by R.A.B Devine [4], the permittivity of amorphous La_2_O_3_ is very low, due to its low density. Therefore, the better crystallinity results in 40%Y (*x* = 0.8) and 70%Y (*x* = 1.4) La_2−*x*_Y*_x_*O_3_ films having a much higher permittivity than La_2_O_3_ film, even though low polarizibility Y^3+^ ions were introduced. Another very important factor is that 40%Y (*x* = 0.8) and 70%Y (*x* = 1.4) La_2−*x*_Y*_x_*O_3_ films were both crystallized in the hexagonal phase rather than the cubic phase. As discussed above, the hexagonal rare earth oxides show much larger permittivities than cubic rare earth oxides as expected from the **Clausius-Mossotti** relationship. It is reasonable that the 40%Y (*x* = 0.8) and 70%Y (*x* = 1.4) La_2−*x*_Y*_x_*O_3_ films which are well crystallized in the hexagonal phase show a high permittivity of 26. In addition, the peak of the hexagonal (002) La_2−*x*_Y*_x_*O_3_ gradually shifts to a larger 2θ as Y concentration increases. This shift is attributed to the decrease of the lattice parameter due to the smaller ionic radius of Y^3+^ than that of La^3+^. For the La_2−*x*_Y*_x_*O_3_ (*x* = 1.8) film, it is found from the XRD pattern that the film contains both the cubic and hexagonal phases. Therefore its permittivity is larger than that of the Y_2_O_3_ with a cubic phase, but smaller than that of 40%Y (*x* = 0.8) and 70%Y (*x* = 1.4) La_2−*x*_Y*_x_*O_3_ films due to the low polarizibility Y^3+^ ion and the low permittivity cubic phase.

**Figure 16:**
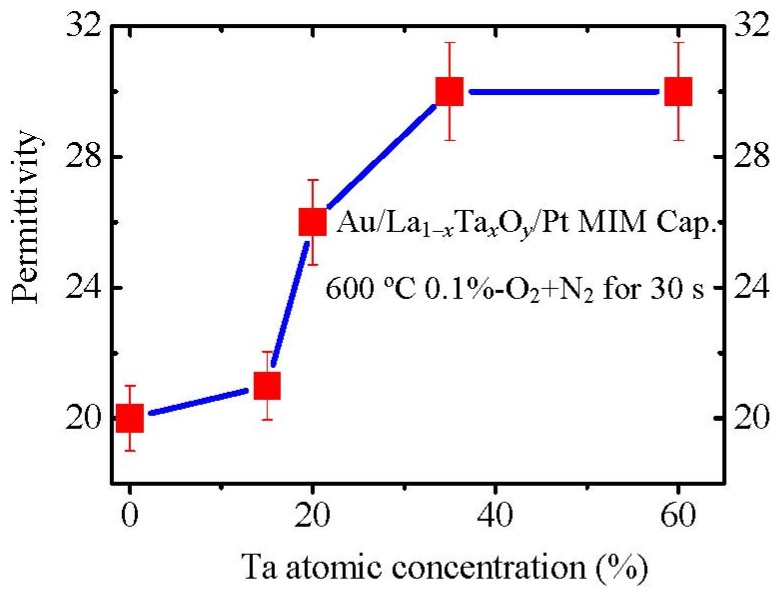
Variation of permittivities of La_1−*x*_Ta*_x_*O*_y_* films with Ta concentration.

We also prepared La_1−*x*_Ta*_x_*O*_y_* films with different Ta concentrations. The permittivities were measured with Au/La_1−*x*_Ta*_x_*O*_y_*/Pt MIM capacitors. La_1−*x*_Ta*_x_*O*_y_*(*x* = 0.35) film shows a high permittivity of about 30 (Figure 16), which is comparable to the largest reported permittivity of La_2_O_3_ [35] and amorphous Ta_2_O_5_. This permittivity value is also much larger than that of amorphous La_1−*x*_Hf*_x_*O*_y_* and well crystallized LaAlO_3_ films. The very possible reason for the high permittivity of amorphous La_1−*x*_Ta*_x_*O*_y_* is a higher density of Ta_2_O_5_ [36,37] than La_2_O_3_. This higher density could induce a higher permittivity. The main reason is as follows: if we assume that the unit structure of La_2_O_3_ is not changed by Ta_2_O_5_ doping, the higher material density would induce a higher dipole density (more dipoles in the unit volume), resulting in a higher permittivity. Therefore, a high density Ta_2_O_5_ doping will enhance the permittivity of La_2_O_3_ although the film is amorphous, which will be discussed in more detail later.

### 3.4. Design of Crystallization Behavior of High-k Gate Dielectric Films

**Figure 17:**
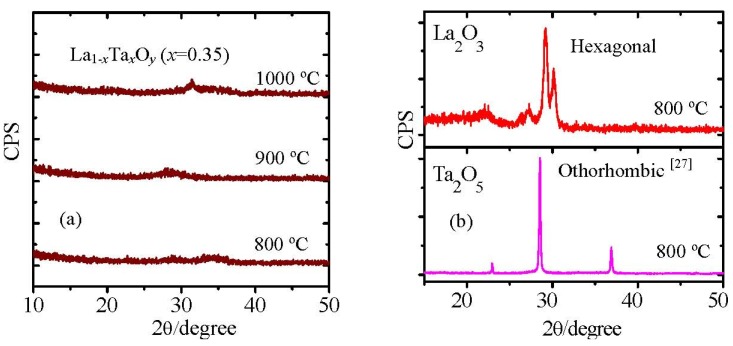
(**a**) XRD patterns of La_1−*x*_Ta*_x_*O*_y_*(*x* = 0.35) film annealed at 800 °C, 900 °C and 1000 °C; (**b**) XRD patterns of La_2_O_3_ and Ta_2_O_5_ films annealed at 800 °C. The thickness of the films was about 30 nm.

Figure 17(a) shows the XRD patterns of La_1−*x*_Ta*_x_*O*_y_* films with a Ta concentration of 35% (*x* = 0.35) after they were annealed at 800 °C, 900 °C and 1000 °C in ambient N_2_. It can be observed that the film was still in the amorphous state even it was annealed at 1000 °C. This indicates that the crystallization temperature of La_1−*x*_Ta*_x_*O*_y_*(*x* = 0.35) film is higher than 1000 °C. As a gate dielectric, an amorphous film is preferred rather than a poly-crystallized film because grain boundaries can induce a leakage current through the dielectric [38]. As La_1−*x*_Ta*_x_*O*_y_*(*x* = 0.35) film shows a crystallization temperature higher than 1,000 °C, it will be compatible with the conventional CMOS process. On the other hand, both La_2_O_3_ and Ta_2_O_5_ films crystallize after annealing at 800 °C (Figure 17b). Furthermore, crystallization temperatures of La_1−*x*_Ta*_x_*O*_y_*(*x* = 0.35) and La_1−*x*_Ta*_x_*O*_y_*(*x* = 0.6) films are about 800 °C and 1000 °C, respectively. Both are higher than that of La_2_O_3_ [39] and Ta_2_O_5_ [35]. It also indicates that the crystallization temperature of La_1−*x*_Ta*_x_*O*_y_* film is sensitive to the Ta concentration, and to prepare the high crystallization temperature La_1−*x*_Ta*_x_*O*_y_* film it is crucial to control the Ta concentration. In the case of La_2−*x*_Y*_x_*O_3_, this shows a very low crystallization temperature [40]. Why is there so large a difference? To answer this question, I will explain the crystallization mechanism of La-based ternary oxides (La_2−*x*_M*_x_*O_3_ or La_1−*x*_M*_x_*O*_y_*) in more detail later. We think there are two main factors to influence the crystallization temperature of La-based ternary oxides. One is the size difference between La and M ions. And the other is the difference of valence state of La and M ions.

It has been reported that the stability of amorphous ternary metal oxide (A_2−*x*_M*_x_*O_3_) is substantially determined by the size difference between metal A ion and metal M ion when A and M has the same valence state [41]. The close size of metal A ion to metal M ion would induce the formation of a solid solution. When there is a large size difference between metal A ion and metal M ion, such a solid solution is not stable. Then, the oxide can be stabilized in the amorphous state. In the case of La-based ternary oxides, the difference between La^3+^ ion size(r(La^3+^)) and M^3+^ ion size(r(M^3+^)) could affect the crystallization temperature of La_2−*x*_M*_x_*O_3_. Table 1 shows the crystallization temperatures of La_2−*x*_Al*_x_*O_3_ [42], La_2−*x*_Sc*_x_*O_3_ [43] and La_2−*x*_Y*_x_*O_3_ films [21]. This is because r(La^3+^) > r(Y^3+^) > r(Sc^3+^) > r(Al^3+^), La_2−*x*_Al*_x_*O_3_ has the highest crystallization temperature, and La_2−*x*_Y*_x_*O_3_ the lowest. Thus, we obtained the well crystallized La_2−*x*_Y*_x_*O_3_ films as discussed earlier.

**Table 1:** Crystallization temperatures of La_1−*x*_M*_x_*O*_y_* ternary metal oxides in the case of M^3+^.

Ternary Oxide (La_2−*x*_M*_x_*O_3_)	Metal Ion Size (La-M)(Å)	Crystallization Temperature (°C)
La_2−*x*_Al*_x_*O_3_	1.16–0.51	800
La_2−*x*_Sc*_x_*O_3_	1.16–0.73	650
La_2−*x*_Y*_x_*O_3_	1.16–1.02	400

We think that the valence state of the M ion can also affect the crystallization temperature of La_1−*x*_M*_x_*O*_y_* ternary oxide because the valence state could determine the oxygen ratio in MO*_y_*. When we dope MO*_y_* into La_2_O_3_ (LaO_1.5_), and if *y* is larger than 1.5, there will be superfluous oxygen which can distort the oxide network and then enhance the crystallization temperature of La_2_O_3_. We studied several La_1−*x*_M*_x_*O*_y_* ternary metal oxides with different valence states of M (Y^3+^, Hf^4+^ and Ta^5+^). The crystallization temperatures of these ternary metal oxides are shown in Table 2. An obvious trend can be found from the table: the ternary metal oxide which exhibits a larger valence state of the M ion (larger *y*) shows a higher crystallization temperature. La_1−*x*_Ta*_x_*O*_y_* ternary oxide shows the highest crystallization temperature among these ternary metal oxides because of the difference of ion size and valence state of La and M. La_2−*x*_Y*_x_*O_3_ films show a low crystallization temperature due to the close size of La^3+^ to Y^3+^ and the same valence state of La^3+^ and Y^3+^. And La_1−*x*_Hf*_x_*O*_y_* film also shows a relatively high crystallization temperature, thanks to the large size difference between La^3+^ and Hf^4+^. In summary, the high crystallization temperature of La_1−*x*_Ta*_x_*O*_y_* film can be attributed to the large size and valence state difference between La^3+^ and Ta^5+^.

**Table 2:** Crystallization temperatures of La_1−*x*_M*_x_*O*_y_* ternary metal oxides in the case of M^3+^, M^4+^ and M^5+^.

Ternary oxide (La_1−*x*_M*_x_*O*_y_*)	Metal ion size(La–M)(Å)	Crystallization temperature (°C)
La_1−*x*_Ta*_x_*O*_y_* (M^5+^)	1.16–0.74	>1000
La_1−*x*_Hf*_x_*O*_y_* (M^4+^)	1.16–0.83	1000
La_2−*x*_Y*_x_*O_3_ (M^3+^)	1.16–1.02	400

### 3.5. Summary

In this paper, most recent progresses of two most important issues, moisture absorption phenomena and low experimental permittivity, of rare earth oxide films used as high-*k* gate insulators for advanced CMOS devices, have been reviewed from both experimental and theoretical points of view.

It has been found that moisture absorption degrades the permittivity of La_2_O_3_ film annealed in N_2_ ambient after exposure to air for several hours because of the formation of La(OH)_3_ with a lower permittivity and it is thus concluded that the moisture absorption could be a possible reason for the scattering *k*-values of La_2_O_3_ films. Furthermore, AFM results indicate that moisture absorption also increases the surface roughness of La_2_O_3_ films on silicon. Thus, an *in situ* gate electrode process would be needed for La_2_O_3_ CMOS application.

Accordingly, the moisture absorption phenomena in main high-*k* gate oxides have been theoretically discussed by comparing the Gibbs free energy change of the moisture absorption reactions of these oxides. The results show that moisture absorption could occur in most high-*k* oxides, especially in rare earth oxides. On the other hand, La_2_O_3_ shows the largest moisture-absorption-reaction rate among main high-*k* oxide candidates. To enhance moisture resistance of La_2_O_3_, doping a second oxide, which has a stronger moisture resistance than La_2_O_3_, could be an applicable solution.

The moisture absorption and associated leakage current of La_2_O_3_ films were suppressed by UV ozone post treatment. The suppression effect by UV ozone treatment has been considered to come from the healing of oxygen vacancies in La_2_O_3_ films, since ambient oxygen annealing also shows the same suppression effect. Compared with ambient oxygen annealing, however, UV ozone post treatment can be carried out at low temperatures, which prevents the formation of a thick interface layer.

With the phase control method, the permittivities and the moisture-resistance of La_2_O_3_ films have been improved significantly. Higher-*k* well crystallized lanthanum based oxide films, La_2−*x*_Y*_x_*O_3_, were prepared, which exhibit a permittivity as high as 28 with an appropriate Y concentration, due to the formation of a high permittivity hexagonal phase, and also show much better resistance to moisture than La_2_O_3_ film after annealing at 600 °C. La_1−*x*_Ta*_x_*O*_y_* films with different Ta concentrations were investigated. The La_1−*x*_Ta*_x_*O*_y_* (*x* = 0.35) film shows not only a high crystallization temperature (>1000 °C), but also a high permittivity (~30).

Furthermore, a systematic discussion on the crystallization behaviors of lanthanum-based ternary oxide has been given, which provides a possible guideline for preparing amorphous or well crystallized lanthanum-based ternary oxides. This should be also useful for other high-*k* oxides to prepare well crystallized or amorphous films as new gate insulators.

## References

[1] Gonzales-Elipe A.R., Espinos J.P., Fernandez A., Munuera G. (1990). XPS study of the surface carbonation/hydroxylation state of metal oxides. Appl. Surf. Sci..

[2] Iwai H., Ohmi S.I., Akama S., Ohshima C., Kikuchi A., Kashiwagi I., Taguchi J., Yamamoto H., Tonotani J., Kim Y. Advanced gate dielectric materials for sub-100 nm CMOS. Proceeding of International Electron Devices Meeting, 2002. IEDM ’02.

[3] Wolf S. (2002). Deep-Submicron Process Technology. Silicon Processing for The VLSI Era.

[4] Devine R.A.B. (2003). Infrared and electrical properties of amorphous sputtered (La_*x*_Al_1–*x*_)_2_O_3_ films. J. Appl. Phys..

[5] Yamada H., Shmizu T., Kurokawa A., Ishii K., Suzuki E. (2003). MOCVD of high-dielectric-constant lanthanum oxide thin films. J. Electrochem. Soc..

[6] Jin H.J., Choi D.J., Kim K.H., Oh K.Y., Hwang C.J. (2003). Effect of structural properties on electrical properties of lanthanum oxide thin film as a gate dielectric. Jpn. J. Appl. Phys..

[7] Shannon R.D. (1993). Dielectric polarizabilities of ions in oxides and fluorides. J. Appl. Phys..

[8] Koehler W.C., Wollan E.O. (1953). Neutron-diffraction study of the structure of the A-form of the rare earth sesquioxides. Acta Crystallogr..

[9] Chin A., Yu Y.H., Chen S.B., Liao C.C., Chen W.J. (2000). High quality La_2_O_3_ and Al_2_O_3_ gate dielectrics with equivalent oxide thickness 5–10 Å. Proceeding of the VLSI Technology, Digest of Technical Papers.

[10] Yokogawa Y., Yoshimura M., Somiya S. (1991). Lattice energy and polymorphism of rare-earth oxides. J. Mater. Sci. Lett..

[11] Kapustinskii A.F. (1956). Lattice energy of ionic crystals. Quart. Rev. Chem. Soc..

[12] Ohni S., Akama S., Kikuchi A., Kashiwagi I., Oshima C., Taguchi J., Yamamoto H., Kobayashi C., Sato K., Tageda M. Rare earth metal oxide gate thin films prepared by E-beam deposition. Proceeding of Extended Abstracts of International Workshop on Gate Insulator, 2001. IWGI 2001.

[13] Mizuno M., Rouanent A., Ymamada T., Noguchi T. (1976). Phase diagram of the system La_2_O_3_-Y_2_O_3_ at high temperature. Yogyo Kyokaishi.

[14] Navrotsky A. (2005). Thermochemical insights into refractory ceramic materials based on oxides with large tetravalent cations. J. Mater. Chem..

[15] Zhao Y., Kita K., Toriumi A. (2010). Thermodynamic analysis of moisture absorption phenomena in high-permittivity oxides as gate dielectrics of advanced complementary-metal-oxide-semiconductor devices. Appl. Phys. Lett..

[16] Mortimer R.G. (2000). Physical Chemistry.

[17] Vasil’ev V.P., Lytkin A.I., Chernyavskaya N.V. (1999). Thermodynamic characteristics of zirconium and hafnium hydroxides in aqueous. J. Therm. Anal. Calorim..

[18] Morant C., Sanz J.M., Galan L., Soriano L., Rueda F. (1993). The O1s x-ray absorption spectra of transition-metal oxides: The TiO_2_-ZrO_2_-HfO_2_ and V_2_O_5_-Nb_2_O_5_-Ta_2_O_5_ series. Surface Sci..

[19] Wilk G.D., Wallace R.M., Anthony J.M. (2001). High-*k* gate dielectrics: Current status and materials properties considerations. J. Appl. Phys..

[20] Zhao Y., Kita K., Kyuno K., Toriumi A. (2009). Band gap enhancement and electrical properties of La_2_O_3_ films doped with Y_2_O_3_ as high-*k* gate insulators. Appl. Phys. Lett..

[21] Zhao Y., Kita K., Kyuno K., Toriumi A. (2006). Higher-*k* LaYO_*x*_ films with strong moisture-resistance. Appl. Phys. Lett..

[22] Zhao Y., Kita K., Kyuno K., Toriumi A. (2007). Suppression of leakage current and moisture absorption of La_2_O_3_ films with ultraviolet ozone post treatment. Jpn. J. Appl. Phys..

[23] Kita K., Kyuno K., Toriumi A. (2009). Origin of electric dipoles formed at high-*k*/SiO_2_ interface. Appl. Phys. Lett..

[24] Song W.J., So S.K., Wang D.Y., Qiu Y., Cao L.L. (2001). Angle dependent X-ray photoemission study on UV-ozone treatments of indium tin oxide. Appl. Surf. Sci..

[25] Zhao Y., Toyama M., Kita K., Kyuno K., Toriumi A. (2006). Moisture-absorption-induced permittivity deterioration and surface roughness enhancement of lanthanum oxide films on silicon. Appl. Phys. Lett..

[26] Wang X.P., Li M.F., Ren C., Yu X.F., Shen C., Ma H.H., Chin A., Zhu C.X., Ning J., Yu M.B. (2006). Tuning effective metal gate work function by a novel gate dielectric HfLaO_*x*_ for nMOSFETs. IEEE Electron Dev. Lett..

[27] Yamamoto Y., Kita K., Kyuno K., Toriumi A. (2006). Structural and electrical properties of HfLaOx films for an amorphous high-*k* gate insulator. Appl. Phys. Lett..

[28] Vellianitis G., Apostolopoulos G., Mavrou G., Argyropoulos K., Dimoulas A., Hooker J.C., Conard T., Butcher M. (2004). MBE lanthanum-based high-*k* gate dielectrics as candidates for SiO_2_ gate oxide replacement. Mater. Sci. Eng. B.

[29] Joshi P.C., Cole M.W. (1999). Influence of post-deposition annealing on the enhanced structural and electrical properties of amorphous and crystalline Ta_2_O_5_ thin films for dynamic random access memory applications. J. Appl. Phys..

[30] Shishido T., Okamura K., Yayima S. (1978). Ln-M-O glasses obtained by rapid quenching using a laser beam. J. Mater. Sci..

[31] Kita K., Kyuno K., Toriumi A. (2005). Permittivity increase of yttrium-doped HfO_2_ through structural phase transformation. Appl. Phys. Lett..

[32] Bottcher C.J.F. (1973). Theory of Electronic Polarization.

[33] Hirosaki N., Ogata S., Kocer C. (2003). *Ab initio* calculation of the crystal structure of the lanthanide Ln_2_O_3_ sesquioxides. J. Alloys Compd..

[34] Coutures J., Rouanent A., Verges R., Foex M. (1976). Etude a haute temperature des systems formes par le sesquioxyde de lanthane et les sesquioxydes de lanthanides. I. Diagrammes de phases (1400 °C <T <T liquide). J. Solid State Chem..

[35] Manchanda L., Morris M.D., Green M.L., Dover R.B., Klemens F., Sorsch T.W., Silverman P.J., Wilk G.D., Busch B., Aravamudhan S. (2001). Multi-component high-*k* gate dielectrics for the silicon industry. Microelectron. Eng..

[36] Pisecny P., Husekova K., Frohlich K., Harmatha L., Soltys J., Machajdik D., Espinos J.P., Jergel M., Jakabovic J. (2004). Growth of lanthanum oxide films for application as a gate dielectric in CMOS technology. Mater. Sci. Semicond. Process..

[37] Abe Y., Kawamura M., Sasaki K. (2005). Oxidation and morphology change of Ru films caused by sputter deposition of Ta_2_O_5_ films. Jpn. J. Appl. Phys..

[38] Ushakov S.V., Brown C.E., Navrotsky A. (2004). Effect of La and Y on crystallization temperature of hafnia and zirconia. J. Mater. Res..

[39] Yajima S., Okayama K., Shishido T. (1973). Glass formation in the Ln-Al-O system. Chem. Lett..

[40] Gusev E.P., Narayanan V., Frank M.M. (2006). Advanced high-*k* dielectric stacks with PolySi and metal gates: Recent progress and current challenges. IBM J. Res. Develop..

[41] Ohmi S., Kobayashi C., Tokumitsu E., Ishiwara H., Iwai H. Low Leakage La_2_O_3_ Gate Insulator Film with EOTs of 0.8~1.2 nm. Proceeding of 2001 Extended Abstracts of International Conference on Solid State Device and Materials (SSDM).

[42] Kakio S., Shimatai Y., Nakagawa Y. (2003). Shear-Horizontal-Type Surface Acoustic Waves on Quartz with Ta_2_O_5_ Thin Film. Jpn. J. Appl. Phys. Part 1.

[43] Li H.J., Price J., Gardner M., Lu N., Kwong D.L. (2006). High permittivity quaternary metal (HfTaTiO_*x*_) oxide layer as an alternative high-*k* gate dielectric. Appl. Phys. Lett..

